# Vaccination Coverage among Prisoners: A Systematic Review

**DOI:** 10.3390/ijerph17207589

**Published:** 2020-10-19

**Authors:** Nancy Vicente-Alcalde, Esther Ruescas-Escolano, Zitta Barrella Harboe, José Tuells

**Affiliations:** 1 Penitentiary Center Alicante II, Carretera N-330, Km. 66, 03400 Villena, Spain; nanvical@gmail.com; 2 Servicio de Urgencias, Hospital Universitario del Vinalopó-Elche, Calle Tonico Sansano Mora, 03293 Elche, Spain; esther.ruescas@gmail.com; 3 Department of Pulmonary and Infectious Diseases, University Hospital of Copenhagen, North Zealand Dyrehavevej 29, 3400 Hillerød, Denmark; Zitta.barrella.harboe@regionh.dk; 4 European Society of Clinical Microbiology and Infectious Diseases, Vaccine Study Group—EVASG, 4010 Basel, Switzerland; 5 Department of Community Nursing, Preventive Medicine and Public Health and History of Science, University of Alicante, San Vicente del Raspeig, 03690 Alicante, Spain

**Keywords:** prisoners, inmates, vaccination coverage, immunization programs

## Abstract

Prison inmates are highly susceptible for several infectious diseases, including vaccine-preventable diseases. We conducted a systematic international literature review on vaccination coverage against hepatitis B virus (HBV), hepatitis A virus (HAV), combined HAV/HBV, tetanus-diphtheria, influenza, pneumococcal, and combined measles, mumps, and rubella (MMR) in prison inmates, according to the PRISMA guidelines. The electronic databases were used Web of Science, MEDLINE, Scopus, and Cinhal. No language or time limit were applied to the search. We defined vaccination coverage as the proportion of vaccinated prisoners. There were no limitations in the search strategy regarding time period or language. Of 1079 identified studies, 28 studies were included in the review. In total, 21 reported on HBV vaccine coverage (range between 16–82%); three on HAV (range between 91–96%); two studies on combined HAV/HBV (77% in the second dose and 58% in the third); three studies on influenza vaccine (range between 36–46%), one of pneumococcal vaccine coverage (12%), and one on MMR coverage (74%). We found that data on vaccination coverage in prison inmates are scarce, heterogeneous, and do not include all relevant vaccines for this group. Current published literature indicate that prison inmates are under-immunized, particularly against HBV, influenza, MMR, and pneumococci. Strengthen immunization programs specifically for this population at risk and improvement of data record systems may contribute to better health care in prisoners.

## 1. Introduction

Today, there are more than 11 million prison inmates around the world [1]. Prison inmates are at higher risk of acquiring communicable diseases compared to the background population [2]. This increased risk is not only related to factors such as overcrowding and high turnover rates of prisoners, but also to factors inherent to the inmates, such as higher proportions of individuals with high-risk behaviors including injecting drug use, sexual risk, and higher prevalence of communicable diseases among others [3,4].

Many prevalent communicable diseases in prisons can be prevented by vaccination. Even though several of these vaccines should be administered as a part of routine national immunization programs during early childhood, factors such as social marginalization, migration, and coming from areas with poor access to health care may contribute to a lower immunization rates, with a higher number of individuals susceptible to vaccine-preventable diseases in prisons around the world [3].

The higher prevalence of communicable diseases among prison inmates may contribute as well to the risk of infectious diseases in the general population since prisoners are often released after short periods of incarceration back into society [5,6,7]. On the other hand, prison inmates belong often to what are considered hard-to-reach populations (e.g., injecting drug users (IDUs), illegal migrants, and sex workers), who may often be reluctant to participate in public health programs [8,9]. Thus, the period of imprisonment can be considered as an opportunity for relevant public health interventions that is often underutilized [10].

Several international health authorities and organizations including the World Health Organization [11], the European Centers for Disease Control [12], and the Centers for Disease Control and Prevention [13] provide a framework with recommendations for the vaccination of prison inmates. A recent review in prisons settings evaluates the acceptance and cost-effectiveness of vaccines, although it is limited in European countries [14].

However, local implementation of these recommendations largely varies across countries; while high-income countries might have specific vaccination guidelines for this group [15,16,17,18], these may be absent or precarious in less industrialized countries. Furthermore, general recommendations may not be very applicable to the reality of prisons in resource-limited settings [4]. 

Estimates of vaccine coverage are one of the main tools that can be used to indirectly estimate the level of immunity against diseases in any given population. Vaccine coverage estimates also reflect the level of adherence to recommendations for immunization and allow us to tailor preventive interventions according to the specific immunization gaps in the population. In this systematic review, we aimed to identify studies on vaccination coverage against several vaccine-preventable diseases in prison inmates and thus identify areas for possible public health interventions. 

## 2. Materials and Methods 

A systematic review of the international literature was carried out following the PRISMA methodology to compile the existing evidence on vaccination coverage in prisons at international level.

The literature search by two reviewers was performed on 20 February 2020 in the electronic databases Web of Science, MEDLINE, Scopus, and Cinhal. The search keywords that were used were: vaccines, vaccination coverage, immunization programs, prisons, prisoners, and inmates in jail. In the search string, the operators “OR” and “AND” were used when relevant, obtaining the following search string: ((((((((“Vaccines”[Mesh]) OR “Vaccines”[Title/Abstract])) OR ((vaccination Coverage[MeSH Terms]) OR vaccination Coverage[Title/Abstract])) OR ((Immunization Programs[MeSH Terms]) OR Immunization Programs[Title/Abstract]))) AND ((((((“Prisons”[Title/Abstract]) OR Prisons[MeSH Terms])) OR ((prisoners[MeSH Terms]) OR prisoners[Title/Abstract])) OR “inmate”[Title/Abstract]) OR “jail”[Title/Abstract])) AND Humans[Mesh]). No language or time limit were applied to the search.

**Figure 1:**
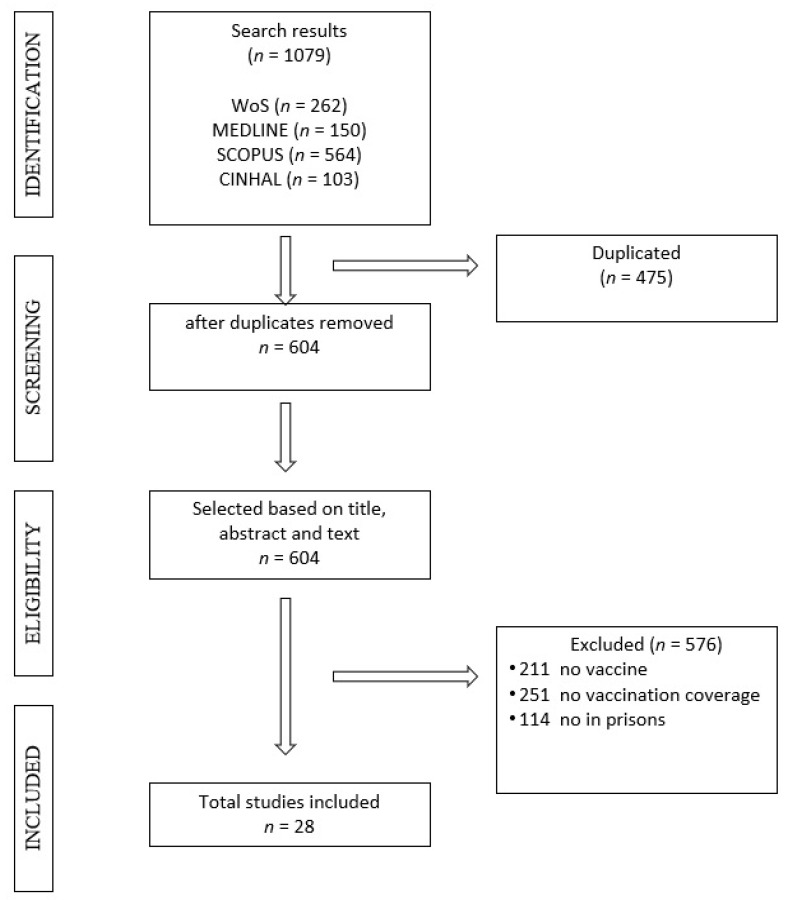
Flowchart selection process (PRISMA).

The following steps were carried out in the bibliographic search: analysis of the documents with extraction of the most relevant information; synthesis of the information that was ordered, combined and evaluated in a comparative way; finally, the end of the search, where we obtained the selected articles included in the review (Figure 1).

The articles were selected through the screening of titles, abstracts and by reading the full text articles, based on predefined inclusion and exclusion criteria based on the PICO (patient, intervention, comparison, outcome) model. Prisoners were defined as any person admitted to prisons. Correctional facilities were defined as jails or prisons and custodial settings that function as prisons, excluding immigrant centers and police detection rooms. The intervention included any strategy that provided data on HIV status and vaccination against communicable diseases upon entry and during prison stay (including outbreak situations). The researchers considered studies with data from community settings, those comparing the inmate population and community settings, those that compared inmates with each other, and those that compared between different risk groups. The following items were considered: people reached by a certain intervention, people who have completed the vaccination, people with serological status, and vaccination coverage. Vaccination coverage was defined as the proportion of prisoners who receive the vaccines recommended by the prison health authorities of their country.

The selected articles had to provide the vaccination coverage of the prisoners regardless of the vaccine studied. Systematic reviews, articles that did not study vaccines, those that did not provide data on vaccination coverage, and those carried out outside the prison setting were excluded. 

All search and selection steps were performed was made by three independent researchers (NVA, ERE, ZBH) and the results were compared and discussed between the three. All selected registries (including articles that raised doubts) were verified by an investigator with experience in the field of vaccine research (JT). The outline of the review can be seen in Figure 1.

Data from selected studies were extracted into tables designed by one investigator and reviewed by a second investigator. The tables contained information on the characteristics of the study: author, year of publication, country (ISO 3166-2), type of study, vaccine studied, methodology for obtaining results (serology, questionnaire, database), type of vaccination carried out (systematic, update of the vaccination program, vaccination of risk groups, or upon admission to prison), vaccination regimen used during the study, study sample, and vaccination coverage.

## 3. Results

Of a total of 1079 results obtained in the four databases consulted, 475 articles were excluded because of duplicates. Subsequently, the remaining 604 articles were reviewed, 576 of which were excluded when applying the inclusion and exclusion criteria, resulting in a final sample of 28 articles (Table 1).

The United Kingdom is the country from which most studies on vaccination coverage in prisoners were retrieved (*n* = 9) [27,28,30,33,34,37,41,44,45], followed by the United States (*n* = 5) [19,22,38,39,40], Australia (*n* = 5) [24,29,31,42,46] and France (*n* = 3) [25,26,32]. To a lesser extent, with one article from each country, we found Spain [21], Italy [35], Luxembourg [36], Canada [41], Switzerland [20], and Denmark [23].

We found the following vaccines and vaccine-preventable diseases studied: hepatitis B virus (HBV), hepatitis A virus (HAV), combined HAV/HBV, influenza, pneumococcal, and combined measles, mumps, and rubella (MMR) vaccines. 

The methods used to investigate the vaccination status of the inmates were: serological testing (*n* = 10) [20,21,23,24,25,35,42,44,45,46], the use of questionnaires (*n* = 5) [28,33,37,38,40], the combination of both (*n* = 3) [26,31,36], the screening of institutional databases (*n* = 5) [27,29,30,32,34], or directly by conducting a survey (*n* = 5) [19,22,39,41,43].

The aim of the studies was to evaluate systematic vaccination or relevant update of the adult vaccination program (*n* = 21) [19,21,22,24,25,26,27,28,29,30,31,32,33,34,35,36,37,38,39,40,41], to explore the coverage of risk groups (*n* = 7) [20,23,36,42,43,44,45], including in some studies vaccination status at the time of admission to prison (*n* = 6) [20,24,35,37,43,46]. The risk groups studied were IDUs and hepatitis C positive (HCV) in the HBV studies [23,42,43,44,45], and men who have sex with men in a combined HAV/HBV immunization study [19] and in another HAV immunization study [20]. In 11 studies (39.2%), exclusively men were included [19,21,25,29,34,35,36,37,39,41,42], in 4 studies (14.2%) only women were included [22,24,28,38], in 11 articles (39.2%) both sexes [23,26,27,30,31,32,33,43,44,45,46] and in 2 (7.1%) these data were not recorded [20,40]. The most studied vaccination coverage was HBV (*n* = 21) [21,22,23,24,25,26,27,28,29,30,31,32,33,34,35,36,42,43,44,45,46] followed by influenza (*n* = 3) [10,29,39], HAV (*n* = 3) [20,36,37], combined HAV/HBV vaccine (*n* = 2) [19,38], pneumococcal (*n* = 1) [29] and MMR (*n* = 1) [41].

Among the 21 studies that focused on HBV vaccine, five studies detailed the reasons why prisoners were not vaccinated [21,22,23,24,25]. As shown in Table 1, most of the studies sought to update HBV vaccination programs to achieve improvements in vaccination coverage [21,22,25,26,27,28,29,30,31,32,33,34]. The vaccination schedules used were different, most commonly at 0 (entry point), 1 month, 2 months, and a booster between 6–12 months. The vaccination coverage obtained ranged between 16% [24] and 82.6% [35]. 

The three studies that provided data on HAV vaccination coverage in prisons are European [20,36,37]. One study was carried out in the United Kingdom as a result of a community outbreak of HAV and included 1363 prisoners in a mass vaccination program and reached a vaccination coverage of 91% [37]. In this study, a similar vaccine coverage (91%) was seen among IDUs [37]. Another study carried out in Luxembourg among 368 prisoners, showed a higher vaccination coverage in non-IDUs (65.9%) than in IDUs (57.1%) [36]. The Swiss study in 2016 included 116 prisoners (52% of African origin) who underwent serological testing that demonstrated immunity in 96% of them [20]. In this study, the authors only recommend HAV vaccination for risk-groups and not routinely for all prisoners.

Two of the studies in our systematic reviewed explored the HAV/HBV combination vaccine [20,38]. The study from Costumbrado et al. [19] published in 2012 focused on a rapid vaccination program in 1633 prisoners (schedule at 0, 7, 21–30 days, and boosted at 12 months) with Twinrix^®^, in men who have sex with men. The coverage achieved was 77% for the second dose, 58% for the third and 11% for the fourth. In the same study, they offered HBV vaccination with the traditional schedule (at 0–1 month and 4–6 months) to all prisoners, with a coverage of 59% for the second dose and 22% for the third dose. The study from Nijhawan et al. [38] published in 2010, carried out on 100 female prisoners in the US, indicated a vaccination coverage for HAV/HBV combined of 47% obtained through surveys.

Gilles and colleagues [29] assessed the coverage of public health interventions in 185 Australian male and female prisoners by accessing local databases. They author found that even though 52% of inmates had at least one chronic disease and an indication for immunization, vaccination coverage for influenza was only 36%, pneumococcal vaccine coverage 12%, and HBV 79%. Robinson et al. [39] studied influenza vaccination in the context of an influenza outbreak identified in two prisons, which led to a mass vaccination campaign of all prisoners. The campaign achieved a vaccination coverage of 42–46% in prisoners and 25–37% in staff. Seib et al. [40] carried out a study using a questionnaire to evaluate vaccination strategies in 25 prisons; they provided data on the vaccination coverage of seasonal influenza of 70% and H1N1 of 64%.

There is only one study reporting on mumps in prisoners in the context of an outbreak in Canada in 2011, in which the MMR vaccine was offered to the entire inmate population. They campaign achieved 83% of vaccination coverage in prisoners and 36% in staff [41].

## 4. Discussion

Vaccination coverage in prison inmates has been a subject of limited interest. Most studies deal with HBV and its direct relationship with a risk group (IDUs or HCV positive inmates) [42,43,44]. In 2016, the World Health Assembly set as a goal eliminating viral hepatitis by 2030, including a 95% reduction in new HBV infections through improved vaccine coverage [47]. The CDC recommends HBV vaccination in prison inmates, considering this population as a high risk-group. The need to achieve adequate HBV immunization levels in high risk populations (e.g., HCV and HIV positive individuals, and IDUs) is widely recognized; however, of the 28 studies included, there were only data from these groups in seven of them. Despite its efficacy and wide availability, HBV vaccine coverage varies substantially by geographic region and prison category, even in high-income countries [30].

Eight studies from the United Kingdom on HBV vaccination coverage have been published, and despite policy changes favoring vaccination in prisons in the 1990s, no major advances have been observed in vaccine coverage [27]. In 2001, new actions were initiated that offered the vaccine against HBV in a limited number of prisons, to all prisoners upon arrival through an accelerated program (0, 1 and 3 weeks, with a booster at 12 months). Since the introduction of this intervention, vaccine coverage has increased: in 2003, the mean HBV vaccine coverage in prisons in England and Wales was 17% [27]; this increased to 22% in 2010 [30]. Perret et al. published data from 2013 where they found coverage of 28.7% and 39.6% in 2017, documenting differences in trends by the type of prison [34].

Regarding the type of interventions, accelerated HBV vaccination programs improved compliance and may contribute to achieve higher immunization rates, particularly for the high proportion of inmates serving short sentences [10,21,23,32,35]. In line with this finding, Palmateer et al. described an association between a higher vaccination coverage in inmates who were imprisoned more than once [45].

The main reasons associated with non-vaccination when carrying out an intervention program in prisons were the release before the administration of the vaccine, previous or current infection, having previously received the vaccine, and refusal to vaccination [21,22,23,24,25,33]. In the Italian study published by Stasi et al., being a foreigner was a risk factor associated with not being vaccinated [35], and in the publication by Taylor et al., the most common reason for not being vaccinated was that inmates never had been offered the vaccine or that they never came to complete the guidelines once they started [33]. 

In this review, a limited number of studies on vaccination coverage of HVA, influenza, pneumococcal, and MMR in prisoners were identified and none on diphtheria-tetanus or BCG were included, similarly to what was published in the review by Madeddu et al. [14] in 2019, focused on vaccination in prisoners of the European Union/European Economic Area.

Even though HAV vaccination of inmates is not routinely recommended by the CDC, vaccination of certain risk groups including, among others, individuals with underling chronic liver diseases, HBV, HCV, and HIV infected individuals, pregnancy, men who have sex with men, and homeless people, all conditions that may be present among inmates [20,37]. HAV immunity in the study by Getaz et al. was high (96%), but likely related to the development immunity during childhood in the regions of origin of inmates. Targeted vaccination according to the area of origin and risk factors for complications (such as chronic liver disease) would improve the immunity of this group and protect vulnerable populations at risk of exposure in this precarious environment in cases of an outbreak of HAV [20].

Few published studies on combined HAV/HBV vaccination were conducted in the US. In a recent review by Madeddu et al. [14] in 2019, there were five studies on this vaccine that were also including cost-effectiveness. 

Regarding influenza, current guidelines recommend offering seasonal influenza vaccine to high-risk groups in the general population, including the elderly and chronically ill, and do not refer to incarcerated persons specifically [48]; except for the Australian study in 2008 [29], which is a study focusing on influenza vaccination in the post-2009 H1N1 pandemic. In the publication by Seib et al. [40] the importance of immunizations in prisons is highlighted given the greater rates of transmission. Despite the experience from the 2009 H1N1 pandemic, influenza vaccines are not routinely administered to prisoners with risk factors, but in contrast initiated in the context of outbreaks.

The only published study on MMR vaccine coverage summarizes the general conclusions of the other studies included in the review: data on vaccine coverage in prisoners are scarce, state vaccination registries are not usually available to verify the vaccination status of prisoners, and inmates are an accessible risk-group [41]. To this should be added the lack of human resources to administer vaccines, especially in emergencies or outbreaks [40].

The most common vaccination modality in a prison is the systematic approach, either as a primary vaccination, as booster or as a post-exposure dose, although massive vaccination programs are also used to control outbreaks. We must remember that, similarly to the general population, acceptance of vaccination is voluntary.

Despite international guidelines and the publication of many evidence-based interventions that demonstrate benefit, a huge gap remains in the introduction and expansion of vaccination programs in prisons in both low- and high-income countries. Cooperation and coordination between the penitentiary and public health systems is necessary to guarantee equity in access to prevention services for the population during incarceration [7].

The extreme sociodemographic heterogeneity of the prisoners, influenced by migration, makes it difficult to assume that the vaccination coverage of this specific population resembles to what otherwise found in the general population at any given time. Carrying out an intervention in closed populations with a large proportion of susceptible individuals exposed to a high risk of contagion, such as the prison community, allows obtaining optimal vaccination coverage and better results compared to vaccination carried out in the general population. This represents an opportunity to approach groups that, under normal conditions, have little contact with the health system. Therefore, it is of special interest to have data on vaccination coverage in prisons in order to prioritize interventions or reinforce vaccination schedules according to the intrinsic risk of each prison; reach as many susceptible people as possible by avoiding vaccinating people who have already been vaccinated or who have already suffered from the disease.

The limitations of the study are mainly those derived from the heterogeneous methodology of the selected studies, since vaccine coverage data are not the main objective of some of them and interpretation of the data was made. This may at least in part explain some discrepancies in the results, when compared to the review by Madeddu et al. [14] Likewise, the heterogeneity in vaccination policies, when they exist, makes studies from different geographical locations not very comparable due to the intrinsic characteristics of each area.

All studies were carried out in industrialized countries, leaving major gaps in the available knowledge we have from prisons in developing countries.

## 5. Conclusions

Prisoners are often an ignored and neglected population regarding public health policies at the international level. Harmonization of recommendations of vaccination strategies in prisons could be a fundamental step to improve access to health care, and for minimizing economic, organizational and disease burden in this setting.

Vaccination strategies varied between countries and were often either targeting a specific vaccine-preventable disease or being implemented as part of an outbreak response. However, subsequent evaluations of the implemented interventions were not carried out systematically or are simply missing. Thus, these approaches may seem arbitrary and we were not able to evaluate whether specific interventions could have contributed to improve vaccination coverage and the overall health conditions of prisoners. 

## Figures and Tables

**Table 1:** Characteristics of included studies.

Author	Year	Country	Design	Vaccine	Metodology	Target Group	Vaccination Schedule	Sample	Coverage
**Costumbrado J. et al.** [19]	2012	US	Cross-sectional	Hepatitis A+B	N	S	0, 7, 21–30 days, 12 months booster	1633 inmates (M)	Dose 2: 77% Dose 3: 58% Booster dose: 11%
**Getaz L. et al.** [20]	2016	CH	Cross-sectional	Hepatitis A	SA	A-RG	single dose	116 inmates (NR)	96%
**Bayas J.M. et al.** [21]	1993	ES	Cross-sectional	Hepatitis B	SA	S	0 months, 15 days–3 months, 5–12 months	705 inmates (M)	Dose 1: 31% Dose 2: 81% Dose 3: 43%
**Clarke J. et al.** [22]	2003	US	Cross-sectional	Hepatitis B	N	S	NR	236 inmates (F)	Dose 1: 67% Dose 2: 48% Dose 3: 27%
**Christensen P.B. et al.** [23]	2004	DK/EE	Open label extension	Hepatitis B	SA	RG	schedule 1: 0, 1, 3 weeks schedule 2: 0 months, 1 months, 6 months	72 DK 566 EE (M/F)	DK: schedule 1: 63% schedule 2: 20% EE: schedule 1: Dose 1: 100% Dose 2: 92% Dose 3: 81%
**Devine A. et al.** [24]	2007	AU	Cross-sectional	Hepatitis B	SA	S-A	0 months, 1 months, 6 months	204 inmates (F)	Dose 1: 83% Dose 2: 40% Dose 3: 16%
**Jacomet C. et al.** [25]	2015	FR	Cross-sectional	Hepatitis B	SA	S	NR	357 inmates (M)	Dose 1: 23% Dose 2: 73% Dose 3: 40%
**Rotily M. et al.** [26]	1997	FR	Cross-sectional	Hepatitis B	SA-QS	S	0 months, 1 months, 2 months	391 inmates (M/F)	Dose 1: 86% Dose 2: 73% Dose 3: 60%
**Gilbert R.L. et al.** [27]	2004	UK	Cross-sectional	Hepatitis B	D	S	>18 years 0, 7, 21 days, 12 months <18 years 0, 1, 2 months, 12 months	42 prisons (M/F)	Average rate 17%
**Plugge E.H. et al.** [28]	2007	UK	Cross-sectional	Hepatitis B	QS	S	NA	613 inmates (F)	27.3%
**Gilles M. et al.** [29]	2008	AU	Cross-sectional	Hepatitis B Influenza Pneumococcal	D	S	NR	185 inmates (M)	Hepatitis B 79% Influenza 36% Pneumococcal: 11%
**Beck CR. et al.** [30]	2012	UK	Retrospective ecological	Hepatitis B	D	S	NR	9173 inmates (M/F)	22%
**Gidding HF. et al.** [31]	2015	AU	Cross-sectional	Hepatitis B	QS-SA	S	0 months, 1 months, 2 months 12 months booster	531 inmates (M/F)	Males: 24.1% Females: 27.3%
**Perrodeau F. et al.** [32]	2016	FR	Cross-sectional	Hepatitis B	D	S	2 doses	231 inmates (M/F)	63%
**Taylor JEB. et al.** [33]	2019	UK	Cross-sectional	Hepatitis B	QS	S	NR	346 inmates(M/F)	52.30%
**Perrett S.E. et al.** [34]	2019	UK	Cross-sectional	Hepatitis B	D	S	0 months, 1 months, 3 months 12 months booster	3560 inmates (M)	2013 28.7% 2017 39.6%
**Stasi C. et al.** [35]	2019	IT	Prospective study	Hepatitis B	SA	S-A	0, 7, 21 days, 12 months	1075 inmates (M)	82.60%
**Removille N. et al.** [36]	2011	LU	Cross-sectional	Hepatitis A+B	QS-SA	S-RG	NR	368 inmates (M)	Hepatitis B IDUs 46.1% nIDUs 37.8% Hepatitis A IDUs 57.1% nIDUs 65.9%
**Gilbert R.L. et al.** [37]	2004	UK	Cross-sectional	Hepatitis A	QS	S-A	single dose	1363 inmates (M)	91%
**Nijhawan A.E. et al.** [38]	2010	US	Cross-sectional	Hepatitis A+B	QS	S	NR	100 inmates (F)	47%
**Robinson S. et al.** [39]	2012	US	Cross-sectional	Influenza	N	S	single dose	Centre A 802 inmates (M) 184 staff Centre B 193 inmates (M) 51 staff	Centre A 42% inmates 37% staff Centre B 46% inmates 25% staff
**Seib K. et al.** [40]	2013	US	Cross-sectional	Influenza	QS	S	single dose	25 correctional (NR)	Seasonal 70% H1N1 64%
**Walkty A. et al.** [41]	2011	CA	Cross-sectional	MMR	N	S	single dose	135 inmates (M) 187 staff	inmates 74% staff 36%
**Awofeso N. et al.** [42]	2001	AU	Cross-sectional	Hepatitis B	SA	RG	0 months, 1 months, 2 months	1037 inmates (M)	1 cohort 85% 2 cohort 79%
**Sutton A.J. et al.** [43]	2006	UK	Model-based	Hepatitis B	N	A-RG	NR	NR (M/F)	5% in 2002 10% in 2003 >50% in 2006
**Hope V.D. et al.** [44]	2007	UK	Prospective survey	Hepatitis B	SA	RG	NR	11,393 inmates (M/F)	1998: 27% 2004: 59%
**Palmateer NE.et al.** [45]	2018	UK	Cross-sectional	Hepatitis B	SA	RG	0 month, 1 month, 2 months 6 months booster	Glasgow prisoners, Scotland prisoners (M/F)	Glasgow inmates 1993–1999 16–20% 2001–2004 52–59% Scotland inmates 2008–2009 71% 2013–2014 77%
**Winter R. et al.** [46]	2016	AU	Cross-sectional	Hepatitis B	SA	A	NR	285 inmates (M/F)	25%

QS: Quest Survey, SA: Serological analysis, D: Database, N: Nothing, S: Systematic/program update, RG: Risk group (IDUs, HIV, HCV), A: Admission. Admission to prison, M: Male, F: Female, NR: no report.
